# Adaptive Strategy for the Statistical Analysis of Connectomes

**DOI:** 10.1371/journal.pone.0023009

**Published:** 2011-08-04

**Authors:** Djalel Eddine Meskaldji, Marie-Christine Ottet, Leila Cammoun, Patric Hagmann, Reto Meuli, Stephan Eliez, Jean Philippe Thiran, Stephan Morgenthaler

**Affiliations:** 1 Signal Processing Laboratory (LTS5), Ecole Polytechnique Fédérale de Lausanne (EPFL), Lausanne, Switzerland; 2 Office Médico-Pédagogique (OMP), Geneva, Switzerland; 3 Departement of Radiology, University Hospital Center and University of Lausanne (CHUV-UNIL), Lausanne, Switzerland; 4 Institute of Mathematics, Ecole Polytechnique Fédérale de Lausanne (EPFL), Lausanne, Switzerland; Newcastle University, United Kingdom

## Abstract

We study an adaptive statistical approach to analyze brain networks represented by brain connection matrices of interregional connectivity (connectomes). Our approach is at a middle level between a global analysis and single connections analysis by considering subnetworks of the global brain network. These subnetworks represent either the inter-connectivity between two brain anatomical regions or by the intra-connectivity within the same brain anatomical region. An appropriate summary statistic, that characterizes a meaningful feature of the subnetwork, is evaluated. Based on this summary statistic, a statistical test is performed to derive the corresponding p-value. The reformulation of the problem in this way reduces the number of statistical tests in an orderly fashion based on our understanding of the problem. Considering the global testing problem, the p-values are corrected to control the rate of false discoveries. Finally, the procedure is followed by a local investigation within the significant subnetworks. We contrast this strategy with the one based on the individual measures in terms of power. We show that this strategy has a great potential, in particular in cases where the subnetworks are well defined and the summary statistics are properly chosen. As an application example, we compare structural brain connection matrices of two groups of subjects with a 22q11.2 deletion syndrome, distinguished by their IQ scores.

## Introduction

Understanding the human brain is one of the greatest challenges in science. A vast number and variety of methods have been developed and applied to analyze and study its organization, development and function. In the recent years, the determination of the interregional brain connectivity (connectome [1], [2]) has attracted much attention given the advent of new in-vivo imaging techniques. One distinguishes between structural connectivity and functional connectivity. Structural connectivity refers to the existence of axonal fibers that interconnect parts of the brain. For example, recent advances in diffusion imaging and tractography permit the construction of high-resolution connection matrices estimating interregional connectivity of the human brain cortex [3]. Functional connectivity is based on joint activation of brain regions and is derived from BOLD contrast MRI, Magnetic EncephaloGraphic (MEG) or other time series data that represent brain activation while the subject performs certain tasks or the subject is in resting state [4], [5]. Structural and functional connection matrices have been used to study properties of brain networks mainly to understand its organization and development [5]–[7]. These connection matrices have also been used to study differences between groups of individuals based on either single connections or by global measures [8]. When performing such group comparisons based on single connections, a large number of correlated statistical tests are routinely performed and, in order to control the occurrences of false discoveries of pair-wise differences, a correction for multiplicity (e.g. Bonferroni or other procedures) is necessary, which greatly reduces the power of the comparisons. We propose in this article a practical and yet effective strategy to analyze complex networks, in particular, brain networks represented by connection matrices. The proposed strategy exploits the potential of high resolution connection matrices by following the concept of “borrowing strength”. In neuroimaging and in many other fields of applications, measures are often correlated and quite well defined regionally. For instance, when analyzing brain networks, instead of performing the statistical tests on the level of single connections between pairs of regions of interest (ROIs), which represent nodes in the brain network, it may be advantageous to reformulate the question in terms of comparisons based on connections between relevant groups of nodes. This is particularly appropriate in cases, where connectivity between groups of ROIs is of major interest to the researcher. The connectivity between groups of ROIs represents a brain subnetwork and corresponds to a block (provided the matrix is properly sorted) in the connection matrix. The proposed strategy is based on the construction of an appropriate statistic in each subnetwork where subnetworks are predefined by the researcher based on prior knowledge. This statistic, which reflects the investigator's research hypothesis or knowledge of the problem, efficiently summarizes a given feature of the subnetwork and will be used for comparisons instead of all the values observed on the ROI level. This strategy has the common advantages of cluster based methods, namely, the reduction of the number of tests and the reduction of the noise variance. Furthermore, the researcher has the opportunity to use some topological network measures that cannot be defined on the single connections level. Of course, the significance obtained by the proposed strategy is at the level of subnetworks and not at the level of single connections. This means that the proposed strategy can be seen as a first stage of an exploratory procedure or coarse scale analysis where the researcher is interested in finding affected subnetworks, that is, those containing one or more affected connections and hence, the interpretation of the results becomes more complex with larger subnetworks. In this direction, we discuss how the proposed strategy could be followed by a local investigation of the connections inside the significant subnetworks.

The paper is organized as follows. First, we briefly discuss the subject of multiple comparisons. Then, we show the benefit of grouping statistical tests into subsets. A particular summary statistic, the mean of values, is studied in detail. We also discuss the possibility of performing a local investigation within the significant subsets. We will call that procedure a “two stage procedure”. After presenting the statistical part of the paper, we show its applicability on complex networks in general and on brain networks in particular. Finally, we present a real application of comparing two groups of structural connection matrices derived from a population of individuals affected with 22q11.2 deletion syndrome [9], [10]. With this application, we will emphasize the structural brain connectivity differences that exist between high (IQ above 70) and low (IQ below 70) cognitive functioning 22q11.2 deletion syndrome [11].

Note that the proposed strategy is applied in this article to brain connection matrices. However, it can be applied to compare and analyze any complex network.

## Materials and Methods

### Multiple comparison procedures

When comparing connection matrices on the level of single connections between pairs of ROIs, the *multiplicity problem* has to be considered. For example, if 10,000 connections are compared simultaneously and if we naively set a level at 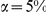 for each single test, we would expect 500 false positives even if no real difference exists. This example shows that it is necessary to control the rate of false positives when multiple comparisons are performed [12]–[14]. The logic of a multiple comparisons situation is summarized in Table 1.

**Table 1:** The general outcome of a multiple comparisons.

Number of hypotheses that are	Statistically non-significant	statistically significant	Total
True	TN	FP	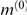
False	FN	TP	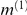
Total	*m-R*	*R*	*m*

A total of *m* null hypotheses are tested. FP is the number of Type I errors or the number of false positives (rejected true hypotheses). Physical significance as indicated in the first column means the existence of a real effect, whereas statistical significance refers to the detection of such effect by means of measurements. FN is the number of Type II errors or the number of false negatives (false hypotheses not rejected). The number *R* of rejected hypotheses is an observable random variable, while FP, FN, TP and TN are unobservable random variables. The number of true null hypotheses 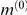 is also unknown in practice. The empirical type I error rate is defined by FP/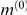, while the empirical type II error rate is defined by FN/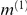 and the estimated average power is TP/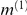. See [12] and [13].

In neuroimaging, most problems involving multiple comparisons control one of two metrics of false positives:

- The Family-Wise Error Rate (FWER): the probability of having at least one false positive (FWER = P(FP>0)).
- The False Discovery Rate (FDR): the expected proportion of false positives among all rejections (FDR = E (FP/*R*) where FP/*R* is defined to be 0 when *R* = 0).

Classical multiple testing procedures control the FWER, which can quite easily be achieved via the Bonferroni procedure. This amounts to dividing the global testing level 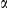 by the number *m* of tests and performing each individual test at that reduced level [15]. The Bonferroni procedure has a very low power, but exerts a strong control over the false positives. As an alternative to the FWER, Benjamini and Hochberg proposed in [12] the FDR and described a procedure that controls it based on Simes' procedure [16]. The FDR has been widely adopted because it increases the power and it is often felt that controlling the FDR is sufficient. Many of procedures that control either the FWER or the FDR are compared in [17]. When the number of tests becomes very large, which is the case in high-resolution neuroimaging, the multiple comparisons procedures are all quite ineffective. However, the advantage, in terms of power, of the FDR procedures, compared to the FWER procedures, becomes more pronounced, but, of course, the expected number of false positives increases with the number of rejections using an FDR control procedure. See [12], [17], [18].

### Grouping methods

Grouping tests or cluster based methods are relying to the concept called “borrowing strength” which is well summarized by [19] who wrote ‘A more explicit use of the dependence structure should result in a powerful method’. This concept was adopted for example in [20] in a geographical application where clusters were defined to be geographical regions. Following this concept, [21] used a cluster based approach to analyze fMRI data to detect activations. They argued by the fact that voxels of a neurological type belonging to a unique anatomical region will usually exhibit positively correlated behavior whether the measure is physiological or functional [21], [22]. A quite different cluster based method was proposed in [23] to analyze fMRI data. In their proposal, the choice of clusters is defined beforehand using prior information. In general, the grouped tests do not have to correspond to units in the same vicinity. For this reason, we say that tests are grouped into subsets instead of using the term of clusters. For, example, a subnetwork could be the connections (edges) between two clusters of ROIs (a bi-subnetwork).

We give a general formulation of grouping tests into subsets, and then explore further the case of using the mean as a summary statistic.

#### General formulation of grouping methods

Consider a set 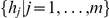 of *m* hypotheses to be tested. Each hypothesis is set to test a certain assessment about a single unit that we call *atom*. The set of all atoms is called the *global set of interest S*. Each single hypothesis 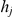 (*j = 1,…,m*) is tested based on the observation of a vector 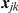 (of dimension *q*) measured in the atom 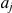 for each individual 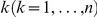. The global data (the design matrix) is of dimension 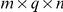. Note that if 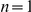, we are reduced to a single-subject experiment. We group the *m* atoms into *s* disjoined subsets 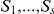 such that 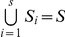 and 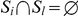 for 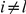, where a subset contains a set of atoms that are linked by attributes associated with the problem at hand. The number of atoms in the subset 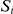 is 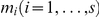, that is, 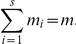
_._

For each subset 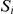 we consider a univariate summary statistic 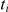, a function of all observed data within the corresponding subset of all individuals, that is 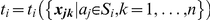 for all 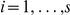. The generalization to a multivariate summary statistic is straightforward. Note that if 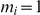, we are reduced to the multivariate atom-wise analysis.

The main advantages of dividing the global set of interest into subsets of atoms are the following: the reduction of the number of tests and the reduction of the noise variance. Effectively, the number of subsets *s* can be much reduced compared to the number *m* of atom-wise tests. For example, using the Bonferroni correction, the global test level α is divided by *s* instead of *m* for each single test. This considerably increases the power of comparisons. In addition, aggregating will reduce the variance of the outcome, which facilitates the detection of significant structures, while isolated significant atoms are rarely considered. This leads to a desirable robustness and is the second reason for increased power. The main disadvantage of grouping methods is the potential loss of information. If the effect is concentrated on a single atom for example, diluting the atom inside a subset weakens the effect. Furthermore, if the subset contains atoms with positive effect and atoms with negative effect, using the mean as a summary statistic clearly disadvantages the grouping methods.

#### Properties of the subset-wise analysis using the mean as a summary statistic

Using the mean of the values observed in all the atoms within a subset is a natural choice of summary statistics. It is also the simplest choice to derive analytical expressions that afford the comparison between the different strategies. However, other summary statistics with a contextual meaning would be preferable such as those we propose in the application to brain networks.

We now, contrast the strategy based on subsets (Subset-Wise-Analysis, SWA) to the approach that separately tests atoms (Atom-Wise-Analysis, AWA) in terms of power. We restrict the comparison to the case of positive effect only.

SWA and AWA solve different problems. While SWA tests the significance of subsets, AWA tests the significance of atoms. This has to be kept in mind when comparing the power of the two different strategies.

Consider the problem of detecting atoms with positive effect when comparing two groups of individuals. Denote by 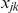 for all *j = 1,…,m* and for all 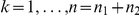
_,_ the measurements associated with the atom 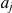 of the individual *k*, where 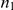 and 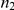 are the sizes of the two groups. For simplification, suppose that 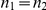 and that the measurements are univariate. For the first group, the observations are of the form 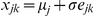 for all *j = 1,…,m* and for all 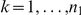. For the second group, in the *non-affected atoms* (where there is no effect), the observed values are of the form 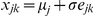 and in the *affected atoms* (that contain the positive effect) the observations are of the form 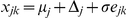 for all 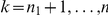. For both cases, 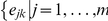 and 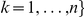 are independent realizations of a normal random white noise, that is, 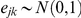. 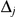 is the raw effect in the atom 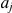. The number of non-affected atoms and affected atoms within the global set of interest are 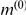 and 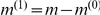 respectively. We assume that the variance 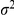 is known and includes both the noise variance and the intra-subject variability. When proceeding according to the AWA to detect the atoms with positive effect, we perform one sided tests 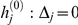 vs. 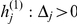, for all *j = 1,…,m*. In all the computations, we use the simple case 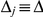.

If the aim is to strongly control the 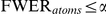 using the Bonferroni procedure, each single test is performed at level 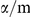. The null hypothesis 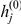 is thus rejected if the difference, 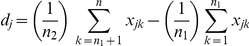 satisfies 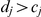, and 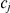 is given by
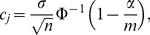
(1)where Φ is the cumulative distribution function of the standard normal distribution.

Now, the global set of interest is divided into *s* subsets. The number of *non-affected subsets*, which contain only non-affected atoms, is 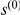 and the number of *affected subsets*, which contain at least one affected atom, is 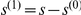. Consider an affected subset of size *m_i_* where only 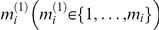 of the atoms are affected with positive effect 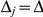. In fact, it is more realistic to consider *partially affected subsets* than considering *completely affected subsets*. This could happen for example if the segmentation that defines the subsets does not exactly match the true limits of the anatomical or functional regions. It could happen also, if only a proportion of atoms in the anatomical or functional region are affected.

For each subset 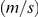 of size *m_i_* 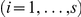, we construct a summary statistic 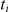 based on the mean of differences between the two groups for each atom in the subset 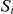, that is, 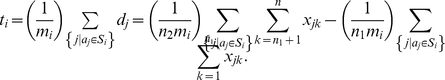


For the non-affected subsets, the statistic 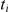
is a realization of a random variable 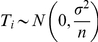, while for the affected subsets, 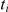 is a realization of a random variable 
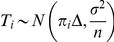
 where 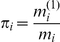 is the proportion of affected atoms in the subset 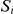. The distribution of 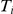 depends on the distribution of 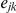. In the non-normal case, if the size 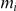 or if the number *n* of subjects are large enough, the central limit theorem (CLT) leads to an approximation of the distribution of 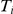 by a normal distribution. Still, we have to consider that the p-values are sensitive to the distribution of the summary statistic in the right tail of the approximated distribution.

We define the 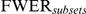 as the probability of having at least one false positive subset, that is, declaring as significant a subset that contains no affected atoms. To control the 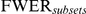 at level α and again, using the Bonferroni procedure on subsets, the null hypothesis 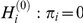 is rejected for the subset 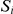 if the corresponding observed summary statistic 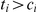, and 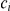 is given by
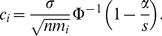
(2)


This relation shows that the critical values for the SWA do not only depend on the ratio 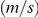 which represents the reduction of the number of tests, but also on 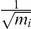 which decreases as long as the subset size increases and is due to the reduction in the noise variance.

We can also compare the power curves corresponding to the two different strategies. In the case of the AWA, the power *Pow_j_* of detecting an affected atom is given by
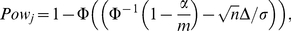
(3)whereas the power of detecting an affected subset that contains a proportion 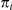 of affected atoms is
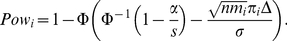
(4)


Given this two power functions, we want to know when 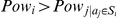, that is, under what conditions we have a greater chance to detect an affected subset 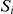 than detecting the affected atoms 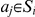. We have
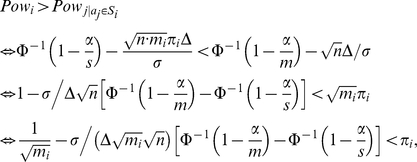
that is, 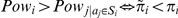, where
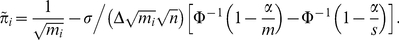
(5)


If the proportion of affected atoms is less than 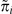, the power of detecting an affected subset is reduced since the effect is diluted. However, when the proportion of affected atoms 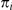 exceeds the threshold value 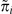, the SWA is more powerful than the AWA in the sense that detecting subsets which contains at least a proportion 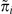 of affected atoms is more powerful than detecting the individual affected atoms within such a subset. The threshold 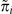 depends on the size of the subset *m_i_*. Larger subsets give more advantage than smaller subsets, but of course, this has an influence only when different resolutions are available since we supposed that the segmentation that defines subsets is predefined. Note that the threshold 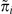 depends also on the variance *σ^2^*, the raw effect Δ and on the number of subjects *n* in the sense that the advantage of the SWA becomes more pronounced when the variance increases (when the Signal to Noise Ratio (SNR) decreases) and when the raw effect and the number of subjects decrease.

Note also that 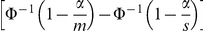 is positive since 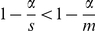 and Φ is an increasing function. Then,
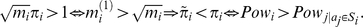
So, a sufficient condition for having 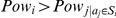 is that the number of affected atoms in that subset is greater than the square root of the size of the subset. A similar result was found by simulations in [23] using an adaptive FDR procedure.

We simulated tests when the observed values in the non-affected atoms and in the affected atoms are independent realizations of 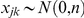 and 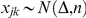 respectively. This is a particular case where 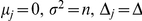. The number of affected atoms 
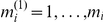
 for all affected subsets. In this case, the power of detecting a significant effect in an affected subset given by equation 4 becomes
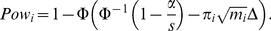
(6)


For simplicity, the *s* subsets are chosen to have the same size 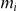, for all *i* in {1,…,*s*}. To control the rate of false positives on atoms and subsets, we used the Bonferroni procedure to control the FWER and the Benjamini-Hochberg procedure (BH95) introduced in [12] to control the FDR.

**Figure 1:**
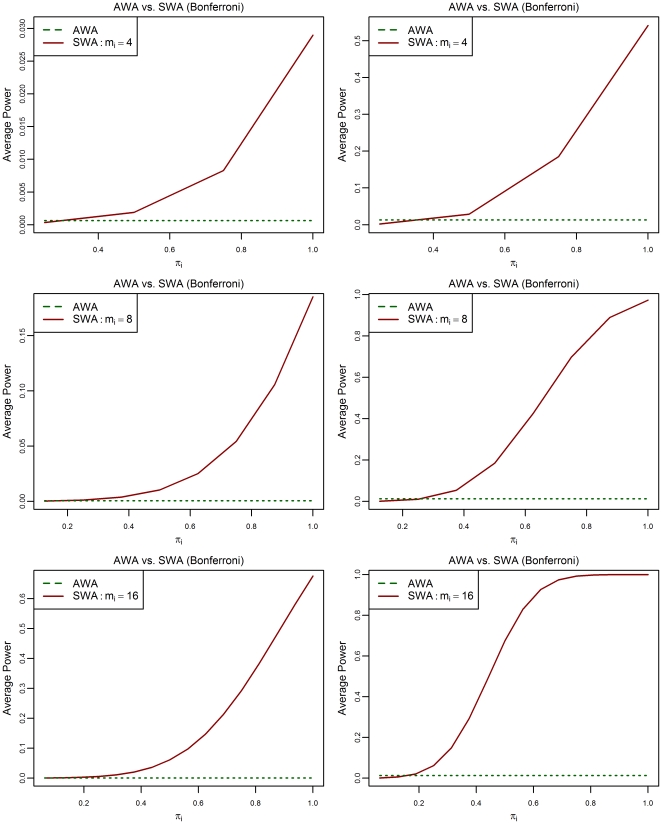
Power of detecting affected atoms and partially affected subsets depending on the proportion 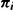. For the multiplicity correction, the Bonferroni procedure is used. Three different values of the subsets' size 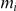 (4, 8 or 16) and two different values of the raw effect Δ (1 or 2). The other parameters are: 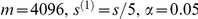.

**Figure 2:**
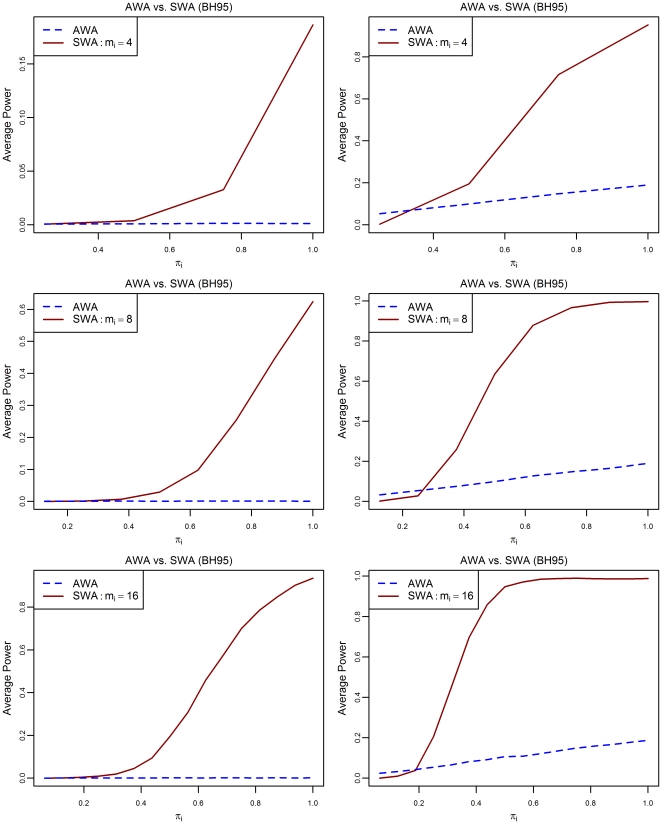
Power of detecting affected atoms and partially affected subsets depending on the proportion 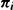. For the multiplicity correction, the BH95 procedure is used. Three different values of the subsets' size 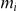 (4, 8 or 16) and two different values of the raw effect Δ (1 or 2) are used. The other parameters are: 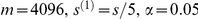.

In Figures 1 and 2, we see the behavior of the power of detecting partially affected subsets, depending on the proportion of truly affected atoms *π_i_* using the SWA, compared to the power of detecting affected atoms using the AWA. In figure 1, we used the Bonferroni procedure, and in Figure 2 we used the BH95 procedure. The raw effect was set to be Δ = 1 or 2 and the subset's sizes are *m_i_ = *4, 8 or 16. The power plotted is the average of 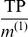 across the simulations (average power defined in [24]). Since 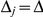 in all affected atoms, the average power is equal to the per-pair power defined in [25]. This holds also for the power of detecting affected subsets since they have the same size, where we obviously have to replace *m* by *s* and 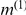 by 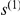. In addition, the power has a different meaning in the two cases. For the AWA we want to detect significance on the atom level, while for the SWA we seek significant subsets.

In all cases, for small values of the proportion 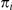, using the mean as a summary statistic reduces the power of detecting a significant effect within such subsets because the effect is diluted. However, when the proportion of affected atoms 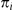 exceeds the threshold value 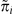, the SWA is more powerful than the AWA in the sense that detecting subsets which contains at least a proportion 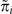 of affected atoms is easier than detecting the individual affected atoms. In both cases, by using the Bonferroni procedure or the BH95 procedure, the threshold 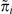 depends on the size of subsets 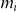 and the raw effect *Δ*. This is in accordance with the result derived analytically for the Bonferroni case.

#### A two-stage atom-wise analysis

In the previous section, we showed that detecting affected subsets using the mean as a summary statistic is more powerful than trying to detect individual affected atoms when a subset contains more than 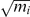 affected atoms. We expect that this condition would be satisfied in neuroimaging, because we assume the existence of positive correlations between atoms within the same subset. This was the principal motivation of our work. We want to shed light in this section, on an interesting question related to the grouping tests strategy. Consider the following two stage AWA procedure. First, we apply the SWA to detect affected subsets. Then, in each subset declared as affected, we perform a local investigation, that is, we apply a multiple comparisons procedure inside that subset to detect affected atoms. Does this have an advantage over the classic AWA procedure? As the question has a complex answer, we restrict our discussion to a simple special case.

#### Proposition 1

The subset 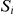 contains 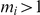 atoms and is considered along with *s* subsets. The corresponding observations are either 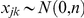 or 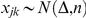. The summary statistic is the subset mean difference 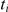, which is significant in the Bonferroni sense, that is, 
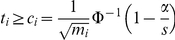
. If 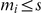, it follows that at least one individual atom will also pass the second-stage Bonferroni test.

#### Proof

Suppose that all atoms in the subset 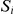 satisfy 
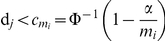
, that is, no atom pass the second-stage Bonferroni test. We have
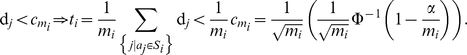



Given that 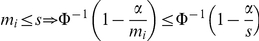, we have
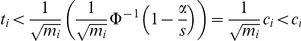



#### Proposition 2

In a significant subset, the power of detecting affected atoms is larger when using the two-stage AWA than using the classic AWA if the Bonferroni procedure is used in both cases.

#### Proof

Obviously this is true, because
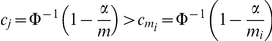
This proposition does not assure that the two-stage AWA is more powerful than the global AWA. In fact, the global AWA may detect other affected atoms, which do not belong to the declared significant subsets. However, the two-stage AWA is more powerful in the subsets that contain more than 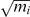. Hence, the two-stage AWA tends to detect affected structures, which is often more interesting for the researcher. See [23].

In addition, simulations show that the rate of the false positives is not controlled in the strong sense by the two-stage AWA even if the Bonferroni procedure is used in the two stages. However, the weak control of false positives can easily be proved in this case.

### The SWA in the context of connection matrices and brain networks

**Figure 3:**
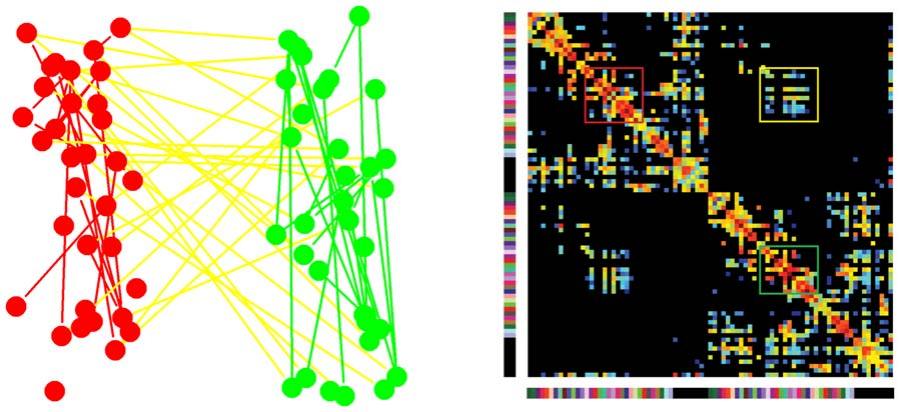
Illustration of the different types of subnetworks within a brain network. In the right side, a connection matrix is presented. In the left side, the connectivity between two groups of node is presented which defines three subnetworks of two types. The first type represents the intra-connection within the same subset of nodes (the red and the green subnetworks) and whose corresponding blocks are localized on the diagonal of the global connection matrix (the red and the green blocks). The second type represents the interconnections between the two subsets of nodes (the yellow subset). Its corresponding block is localized out of the diagonal in the global connection matrix (the yellow block).

Suppose that a global region of interest in the human brain is subdivided into small ROIs. A connection matrix is a weighted symmetric matrix *A* where rows/columns correspond to ROIs and each cell *A(r,r')* of the matrix represents a certain measure of the connectivity between the two ROIs *r* and *r'*. A connection matrix defines a network where the nodes correspond to the ROIs and the weighted edges correspond to the measure of the connectivity between the corresponding ROIs. A subnetwork of the human brain network corresponds to a block of the connection matrix. We consider separately two kinds of subnetworks. The first type represents the intra-connection within the same group of ROIs and whose corresponding blocks are localized on the diagonal of the global connection matrix. The second type corresponds to the networks that represent the interconnections between two groups of ROIs. These are bi-subnetworks and their corresponding blocks are localized out of the diagonal in the global connection matrix. See Figure 3 for an illustration.

#### How to choose subsets or subnetworks

As mentioned in the introduction, the subsets or subnetworks are chosen beforehand by the researcher based on an a priori knowledge. This prior information is obtained either by an investigation of independent data sets or by using a segmentation atlas. Both the AWA and the SWA are hampered by a common difficulty, due to the fact that atoms or subsets have to correspond between the different subjects. This important difficulty was emphasized in [23]. In practice, the ROIs do not exactly match geometrically between individuals, in particular for higher resolutions. Consequently, the effect of affected atoms will be scattered and will be diluted. However, the mismatched ROIs have more chance to be located in the same group of ROIs used to define a subnetwork, in particular, if they are geometrically not close to the frontiers of the subregions. In this case, the advantage of the SWA is even more pronounced. The mismatching between atoms causes problems for any method that define subsets on the basis of the estimated correlations between atoms.

#### Summary statistics and statistical analysis

As mentioned in the introduction, three levels of analysis are considered to analyze the brain complex network. The first level is the atom level where we perform an AWA. Atoms could be an ROI (a node) or a connection between two ROIs (an edge). In the first case, one could use some network measures that can be evaluated for a node. For example, one can evaluate for each node its degree, its clustering coefficient, etc. In the second case, the weight of the edge can be used. We call the AWA performed with network based measures the Atom Network Based Analysis (ANBA). The second level considered is the global level that corresponds to the global network. We call the analysis at that level the Global Network Based Analysis (GNBA). In the third level, we consider the analysis at the level of subnetworks by considering each subnetwork as a complex network. We call the analysis at that level the Sub-Network Based Analysis (SNBA). In the two last levels, a variety of summary statistics could be chosen, but of course, the number of tests performed is not the same at the two levels. The choice of summary statistics depends on the subnetwork. One can construct summary statistics by averaging the network based measures on atoms within each subnetwork. This has the properties of the subset mean discussed in the previous sections. For example, one can use the mean of the edges' weights or the mean of the nodes' degrees, centrality, efficiency, modularity, etc. On the other hand, several summary statistics which do not necessarily use the mean of the values and which cannot be defined on the level of atoms could be used as a summary statistic. For example, one can use the node-degree distribution, the small world properties, etc. of each sub-network as a summary statistic. See [26], [27] for a list of relevant network measures that could be used in the brain network analysis. It is difficult to use network based summary statistics that do not use the mean of atom values such as small world properties, as a summary statistic since the control of false positives depends on the distribution of the summary statistics, in particular, when using the two-stage procedure, where conditional distributions come into play. In addition, the network based measures are evaluated on noisy connection matrices. It is then, worth to investigate the impact of this factor on the statistical analysis. This two important aspects need to be investigated and they are parts of our future work.

### Application to structural connection matrices of the human brain

The purpose of this application section is to give a simple example of a real application of the proposed strategy to compare normalized whole-brain structural connection matrices derived from diffusion MRI tractography.

#### The construction of structural brain connection matrices

The processing pipeline used to derive connection matrices compared in this application is basically divided into two pathways. See [3] for more details. On one hand, the cortical surface is extracted from a high resolution T1-weighted Magnetic Resonance (MR) image and subdivided into 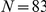 anatomical parcels by matching the most important sulci using atlas-based segmentation. On the other hand, a whole brain tractography is performed on diffusion MR images, which results in millions of virtual fibers spread over the brain. The combination of these two procedures allows the construction of connection matrices by computing the connection density for each pair of ROIs. Considering the cortical parcellation and the white matter tractography described above, the fiber bundle *F(r,r')* connecting the pair of ROIs *(r,r')* could be identified. The value of the connection matrix cell *A(r,r')* is the connection density between these ROIs and is defined as follows: 
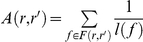
, where *l(f)* is the length of the fiber *f* along its trajectory.

**Figure 4:**
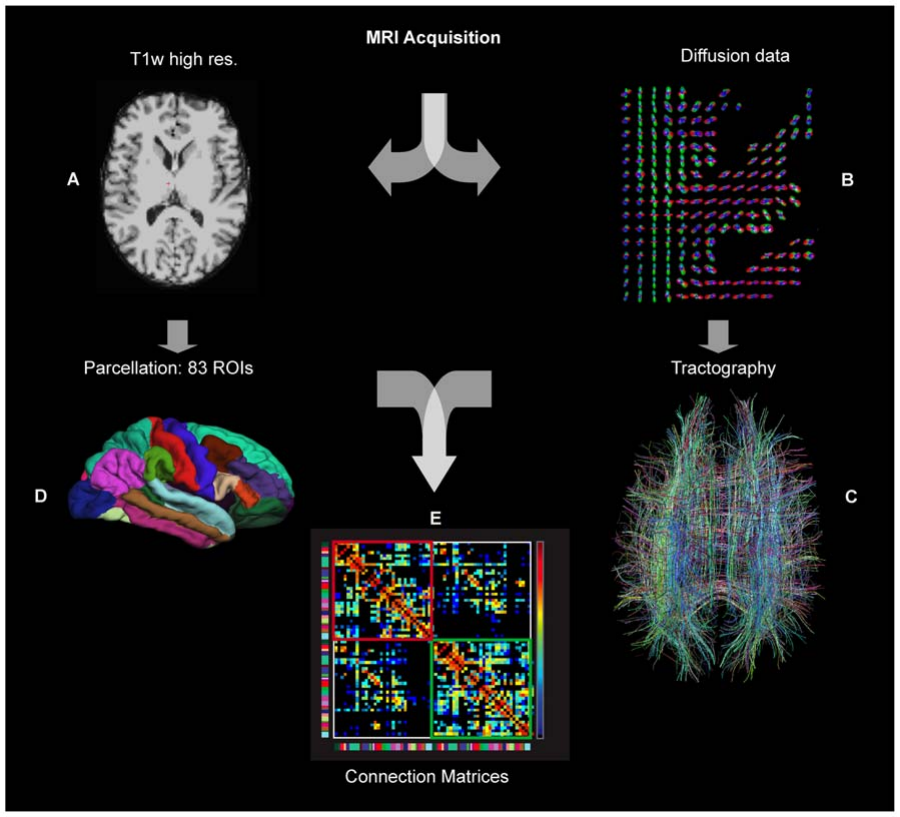
Extraction of a Whole Brain Structural Connection Matrix. **A–B.** MRI Acquisition: (A) high-resolution T1-weighted image and (B) diffusion images. The T1 is registered on the diffusion images. In every imaged voxel the Orientation Density Function (ODF) is extracted from the diffusion images. **C.** Whole brain tractography provides an estimate of axonal trajectories across the WM. **D.** Cortex partitioning into 83 gyral-based parcels using the Freesurfer software (http://surfer.nmr.mgh.harvard.edu). **E.** Creation of the low-resolution structural connection matrix, representing the fiber density between every pair of the 83 parcels (upper left and lower right blocks: connections in the right, respectively left hemisphere; off-diagonal blocks: inter-hemispheric connections).

The correction term *l(f)* in the denominator is needed to eliminate the linear bias towards longer fibers introduced by the tractography algorithm. We obtain at the end of the application of the pipeline an *N×N* symmetric matrix *A*. The pipeline is summarized in Figure 4.

#### Description of the data

The clinical group used for comparing the AWA and the SWA or equivalently, for comparing ANBA and SNBA, is a group of subjects with a 22q11.2 deletion syndrome (22q11DS) [9], [10]. Amongst other manifestations, this syndrome shows a mild cognitive impairment (MCI) resulting from a loss of IQ performance [11], [28].

Amongst the 22q11DS population, there is an existing discrepancy in the cognitive abilities between patients with a relatively high IQ level (above 70) and patients with a low IQ level (below 70). Delineating the structural brain connectivity that sustains this discrepancy may provide useful clues to understand how brain connections are involved in the loss of intellectual functioning in the 22q11DS population.

The high IQ group (>70) is composed of 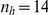 patients, 7 girls and 7 boys (mean age = 14.5±2.9 years, ranged from 7.4 to 17.6 years and mean IQ = 80.4±6.7). The low IQ group (<70) is composed of 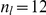 patients, 5 girls and 7 boys (mean age 14.8±3.9 years old ranged from 7.2 to 19.8 years and mean IQ = 60±6.8).

In addition of the connection density matrices, we have Fractional Anisotropy (FA) connection matrices where the weight represents the mean FA along a fiber tract connecting a pair of ROIs.

#### Data analysis

To detect atoms (connections) or subnetworks that differ between the two groups, we consider different strategies:

First, we consider the ANBA analysis using all the available variables (density of fibers, FA, density of fibers truncated by the FA) and the combinations of variables as univariate and multivariate statistics.

Second, we consider the SNBA analysis with different summary statistics.

- The SWA using the mean of the density of fibers 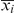 in each subnetwork as a summary statistic.
- The truncated proportion, where the summary statistic in each subnetwork is defined by 
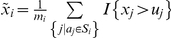
, where *I* is the indicator function. This variable is interpreted as the number of interconnections between ROIs within the subnetwork 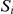 (number of edges). We use here, a truncation threshold based on the FA values. One can choose other thresholds for the truncation depending on the nature of the problem.
- The effective mean, where the summary statistic is defined by 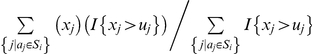 which is interpreted as the mean of edges' weights in the subnetwork 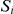. Since the distribution of the two later statistics is unknown, we use the Wilcoxon-Mann-Whitney rank test [29] instead of the Student test to derive the p-values.
- The summary ***t*** statistic is a p-variate statistic that includes a combination of *p* univariate statistics among the summary statistics defined above. In this case, the test follows by computing the statistic *f*: 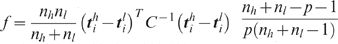, where *C* is the estimated covariance matrix of the data given by 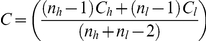, where 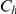 and 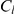 are the estimated covariance matrices of the high IQ group and the low IQ group respectively. The statistic *f* follows a Fisher distribution 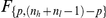.

We apply the SNBA to the data by defining subnetworks as the interconnections between brain cortex lobes. The brain cortex is divided into 13 lobes. By considering the interconnections between these 13 lobes and the intra-connections within these 13 lobes, we define 
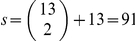
 subnetworks. The SNBA is followed by a local investigation in the subnetworks detected as significant.

For the multiplicity correction of p-values we use the Bonferroni correction with level FWER = 0.05 and the BH95 procedure with FDR = 0.1 for all tests.

## Results and Discussion

First, using the AWA (or the ANBA), no significant results were found with any statistic (univariate or multivariate). This is due first, to the small number of individuals in each group and second, to the fact that the two compared groups belong to the same community of patients and so, no big differences could exist between the two groups (small raw effect) and of course, this is due to the multiplicity correction since 
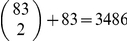
 tests are performed simultaneously.

Using the SWA (or the SNBA), three subnetworks were detected as significant after the BH95 correction of the p-values. Only one of the three subnetworks passed the Bonferroni correction.

Within these three subnetworks, only 5 connections are detected as significantly different within the three detected subnetworks.

The application results presented here are in accordance with the performance showed in the theoretical part either by analytical formulas or by simulations. We are in a situation where the raw effect and the sample size are both small. So, the advantage of the SWA over the AWA is pronounced.

Amongst the five significant connections detected as significant, four were found to be connectively reduced in 22q11DS patients with a low IQ compared with the ones with a high IQ. Right caudate intra-connectivity and the number of fibers connecting it with the putamen were reduced. Right accumbens nucleus inter-connectivity with sub-thalamic nucleus, and left lingual intra-connectivity were also found reduced. The last connection referring to the number of fibers connecting the left putamen and the left superior temporal cortex were increased.

Morphological alterations of the caudate, putamen, left superior temporal gyrus (STG) and lingual area have frequently been found in 22q11DS [30]–[32].

Here, we show that there is a specific alteration of the connectivity of the striatal structure (composed of the caudate and the putamen) affecting the cortico-striatonigral-thalamocortical circuit [33] and therefore may impairs the cognitive functioning [34] in the 22q11DS with the low IQ.

### Conclusion and future directions

We proposed a statistical network based strategy to analyze and compare brain networks with the aim of increasing the power of detections. The strategy is based on grouping tests into subsets that define brain subnetworks in order to reduce the number of tests. We showed in the simulation examples and in the real application the relevance in neuroimaging, in particular, when the number of tests is very large and when the raw effect and the sample size are small. We proposed as well the use of the local investigation in the significant subnetworks in a second stage. We can summarize the subnetwork based analysis (SNBA) as follows:

1. Define subnetworks based on the prior knowledge.
2. Choose an appropriate summary statistic for each subnetwork, which has a contextual interpretation.
3. Apply a multiple comparisons procedure (that controls the FWER, the FDR or other measures).
4. If desired, a local investigation in significant subnetworks may help to interpret the results.

We showed that if a subset of size 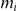 contains more than 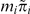 affected atoms where 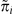 is given by equation 5, the power of detecting such a subset is greater than the power of detecting each affected atom within the subset. The threshold 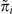 is always smaller than 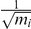 and we expect that this condition i.e. (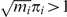) is often satisfied in neuroimaging, that is, a part of a subset or a subnetwork behaves coherently.

It should be emphasized that the proposed strategy as presented in the application uses some particular examples of summary statistics and shows a real advantage over the atom-wise comparisons. A part of our future work will be focused on how to estimate more complex network based summary statistics on noisy connection matrices and to show the control of false positives under their use.
